# *catena*-Poly[[(ethanol-κ*O*)sodium(I)]-di-μ-aqua-[(*rac*-2′-hydr­oxy-1,1′-binaphthyl-2-yl phosphato-κ*O*)sodium]-tri-μ-aqua]

**DOI:** 10.1107/S1600536809021394

**Published:** 2009-06-10

**Authors:** Yuya Tachibana, Yasukatsu Maeda

**Affiliations:** a Industrial Technology Center of Wakayama Prefecture, 60 Ogura, Wakayama 649-6261, Japan

## Abstract

The asymmetric unit of the polymeric title compound, [Na_2_(C_20_H_13_O_5_P)(C_2_H_6_O)(H_2_O)_5_]_*n*_, consists of two Na^I^ ions, one 2′-hydr­oxy-1,1′-binaphthyl-2-yl phosphate anion, one ethanol ligand and  five water molecules of crysallization. Each Na^I^ ion has a distorted octa­hedral coordination geometry. The phosphate anion coordinates to one Na^I^ ion and the ethanol mol­ecule coordinates to the other. The five water mol­ecules bridge the Na^I^ ions, forming an inorganic chain structure along the *b* axis. The chains are connected by O—H⋯O hydrogen bonds into an organic–inorganic hybrid layer parallel to (001).

## Related literature

For organic–inorganic hybrid materials, see: Eckert & Ward (2001 ▶). For phosphate derivatives, see: Vioux *et al.* (2004 ▶).
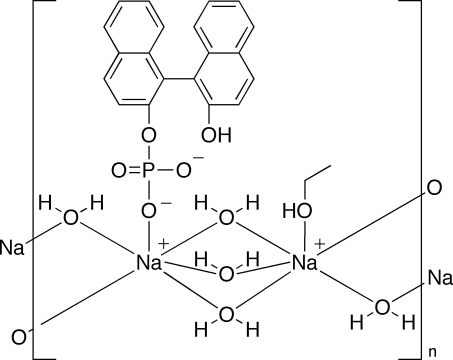


## Experimental

### 

#### Crystal data

[Na_2_(C_20_H_13_O_5_P)(C_2_H_6_O)(H_2_O)_5_]
*M* *_r_* = 546.40
Monoclinic, 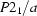

*a* = 13.121 (4) Å
*b* = 9.816 (3) Å
*c* = 20.198 (7) Å
β = 100.033 (13)°
*V* = 2561.7 (14) Å^3^
*Z* = 4
Mo *K*α radiation
μ = 0.20 mm^−1^
*T* = 293 K
0.60 × 0.50 × 0.10 mm

#### Data collection

Rigaku R-AXIS RAPID diffractometer
Absorption correction: multi-scan (**ABSCOR**; Higashi, 1995 ▶) *T*_min_ = 0.874, *T*_max_ = 0.980
24950 measured reflections
5829 independent reflections
5045 reflections with *I* > 2σ(*I*)
*R*_int_ = 0.035

#### Refinement

*R*[*F* ^2^ > 2σ(*F* ^2^)] = 0.037
*wR*(*F* ^2^) = 0.114
*S* = 1.05
5829 reflections
326 parameters
12 restraints
H atoms treated by a mixture of independent and constrained refinement
Δρ_max_ = 0.36 e Å^−3^
Δρ_min_ = −0.46 e Å^−3^

### 

Data collection: *PROCESS-AUTO* (Rigaku, 1998 ▶); cell refinement: *PROCESS-AUTO*; data reduction: *CrystalStructure* (Rigaku/MSC, 2004 ▶); program(s) used to solve structure: *SIR92* (Altomare *et al.*, 1994 ▶); program(s) used to refine structure: *SHELXL97* (Sheldrick, 2008 ▶); molecular graphics: *ORTEP-3 for Windows* (Farrugia, 1997 ▶); software used to prepare material for publication: *CrystalStructure*.

## Supplementary Material

Crystal structure: contains datablocks glogal, I. DOI: 10.1107/S1600536809021394/is2427sup1.cif

Structure factors: contains datablocks I. DOI: 10.1107/S1600536809021394/is2427Isup2.hkl

Additional supplementary materials:  crystallographic information; 3D view; checkCIF report

## Figures and Tables

**Table 1:** Hydrogen-bond geometry (Å, °)

*D*—H⋯*A*	*D*—H	H⋯*A*	*D*⋯*A*	*D*—H⋯*A*
O1—H1⋯O4	0.847 (17)	1.839 (17)	2.6687 (19)	166.3 (18)
O6—H2⋯O11^i^	0.852 (16)	2.043 (17)	2.854 (2)	159.0 (16)
O6—H3⋯O9^i^	0.848 (16)	1.956 (16)	2.7958 (19)	170.5 (16)
O7—H4⋯O5^ii^	0.853 (11)	1.873 (10)	2.7136 (18)	168.4 (19)
O7—H5⋯O4^i^	0.851 (14)	2.106 (14)	2.9473 (19)	169.9 (18)
O8—H6⋯O1^i^	0.847 (16)	1.955 (16)	2.775 (2)	162.7 (16)
O9—H8⋯O4	0.852 (15)	1.875 (15)	2.7259 (18)	177.5 (12)
O9—H9⋯O3^iii^	0.851 (12)	1.850 (12)	2.6978 (18)	174.1 (16)
O10—H10⋯O5^iv^	0.850 (15)	2.197 (15)	3.012 (2)	161 (2)
O10—H11⋯O3^i^	0.849 (12)	2.197 (9)	2.984 (2)	154.1 (19)
O11—H12⋯O3^iv^	0.851 (9)	1.823 (11)	2.6711 (19)	174 (2)

Symmetry codes: (i) 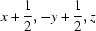; (ii) 
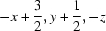
; (iii) 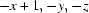; (iv) 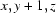.

**Fig. 1.**
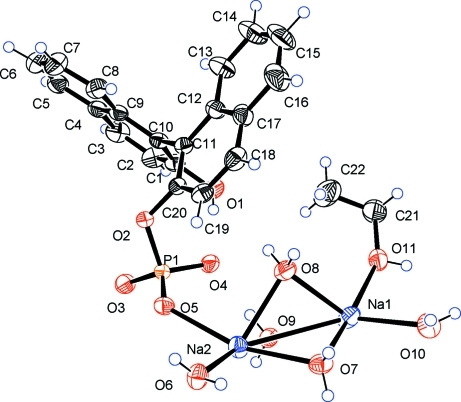
A view of the title compound, showing 50% probability displacement ellipsoids.

**Fig. 2.**
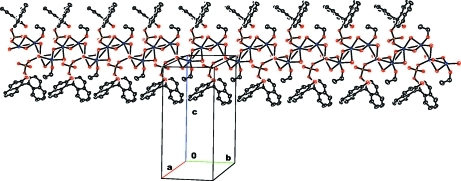
The one dimensional chain structure of the title compound bridged between sodium cations and water molecules. Hydrogen atoms have been omitted for clarity.

**Fig. 3.**
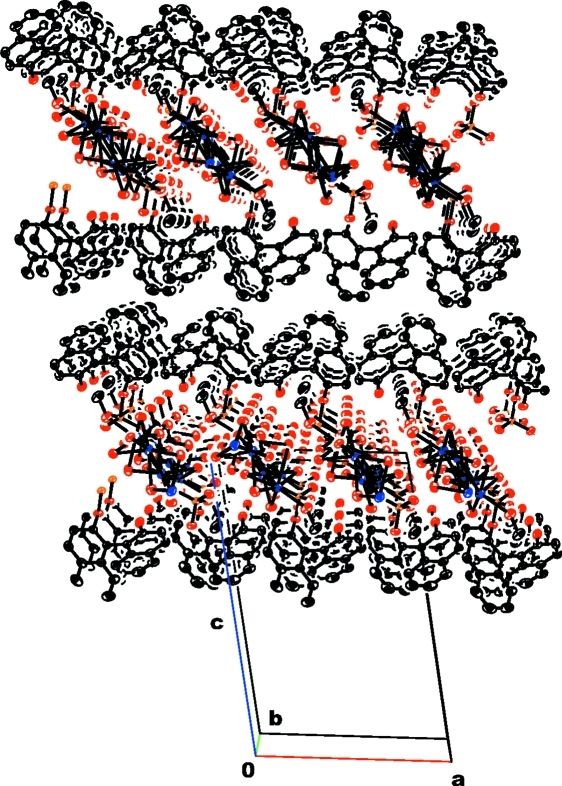
The layered structure of the title compound. Hydrogen atoms have been omitted for clarity.

## supplementary crystallographic information

### Comment

In recent years, studies on organic-inorganic hybrid layered materials have received a great deal of attention as electrical, magnetic and/or optical materials (Eckert & Ward, 2001). Some combinations are studied to form layered structure. Phosphate ester can form organic-inorganic hybrid network (Vioux *et al.*, 2004).

The title compound has inorganic layer formed with two sodium cation, phosphate anion and five waters, and organic layer formed by binaphthyl unit. The phosphate anion coordinates to one  Na^I^ atom and the ethanol molecule coordinates to the other Na^I^ atom as shown in Fig. 1. Sodium ions in the compound are bridged by three water molecules, and one sodium ion connected to adjoining sodium ion through two water molecules to form a one-dimensional zigzag chain structure along the *b* axis as shown in Fig. 2. The chains build two-dimensional network along the *a-b* plane by hydrogen bonding, and form inorganic layer. Organic layers, build up with binaphthyl moiety, and inorganic layers form bilayered structure in the crystal. Then, three-dimensional organic-inorganic hybrid layered material is formed by self-assembly as shown in Fig. 3.

### Experimental

Sodium hydride (1.5 g, 63 mmol) was dispersed into tetrahydrofuran (50 ml). (*rac*)-1,1'-Binaphtyl-2,2'-diyl hydrogenphosphate (1.0 g, 2.9 mmol) was added to the dispersion and stirred for 2 h at room temperature. The resulting mixture was filtrated and evaporated *in vacuo*. The residue was recrystallized from H_2_O/ethanol.

### Refinement

All C-bound H atoms were positioned geometrically (C—H = 0.93–0.97 Å) and refined as riding atoms, with *U*_iso_(H) = 1.2*U*_eq_(C) or 1.5*U*_eq_(methyl C). O-bound H atoms were located in a difference Fourier map and the positional parameters were refined, with distance restraints of O—H = 0.850 (5) Å, and with *U*_iso_(H) = 1.2*U*_eq_(O).

### Crystal data

**Table:**

[Na_2_(C_20_H_13_O_5_P)(C_2_H_6_O)(H_2_O)_5_]	*F*(000) = 1144
*M**_r_* = 546.40	*D*_x_ = 1.417 Mg m^−^^3^
Monoclinic, *P*2_1_/*a*	Mo *K*α radiation, λ = 0.71075 Å
Hall symbol: -P 2yab	Cell parameters from 19717 reflections
*a* = 13.121 (4) Å	θ = 3.1–27.5°
*b* = 9.816 (3) Å	µ = 0.20 mm^−^^1^
*c* = 20.198 (7) Å	*T* = 293 K
β = 100.033 (13)°	Platelet, colourless
*V* = 2561.7 (14) Å^3^	0.60 × 0.50 × 0.10 mm
*Z* = 4	

### Data collection

**Table:**

Rigaku R-AXIS RAPID diffractometer	5829 independent reflections
Radiation source: fine-focus sealed tube	5045 reflections with *I* > 2σ(*I*)
graphite	*R*_int_ = 0.035
Detector resolution: 5.00 pixels mm^-1^	θ_max_ = 27.5°, θ_min_ = 3.1°
ω scans	*h* = −16→16
Absorption correction: multi-scan (*ABSCOR*; Higashi, 1995)	*k* = −12→12
*T*_min_ = 0.874, *T*_max_ = 0.980	*l* = −26→26
24950 measured reflections	

### Refinement

**Table:**

Refinement on *F*^2^	Primary atom site location: structure-invariant direct methods
Least-squares matrix: full	Secondary atom site location: difference Fourier map
*R*[*F*^2^ > 2σ(*F*^2^)] = 0.037	Hydrogen site location: inferred from neighbouring sites
*wR*(*F*^2^) = 0.114	H atoms treated by a mixture of independent and constrained refinement
*S* = 1.05	*w* = 1/[σ^2^(*F*_o_^2^) + (0.0682*P*)^2^ + 0.7208*P*] where *P* = (*F*_o_^2^ + 2*F*_c_^2^)/3
5829 reflections	(Δ/σ)_max_ = 0.006
326 parameters	Δρ_max_ = 0.36 e Å^−^^3^
12 restraints	Δρ_min_ = −0.46 e Å^−^^3^

### Special details

**Table:**

Geometry. All e.s.d.'s (except the e.s.d. in the dihedral angle between two l.s. planes) are estimated using the full covariance matrix. The cell e.s.d.'s are taken into account individually in the estimation of e.s.d.'s in distances, angles and torsion angles; correlations between e.s.d.'s in cell parameters are only used when they are defined by crystal symmetry. An approximate (isotropic) treatment of cell e.s.d.'s is used for estimating e.s.d.'s involving l.s. planes.
Refinement. Refinement of *F*^2^ against ALL reflections. The weighted *R*-factor *wR* and goodness of fit *S* are based on *F*^2^, conventional *R*-factors *R* are based on *F*, with *F* set to zero for negative *F*^2^. The threshold expression of *F*^2^ > σ(*F*^2^) is used only for calculating *R*-factors(gt) *etc*. and is not relevant to the choice of reflections for refinement. *R*-factors based on *F*^2^ are statistically about twice as large as those based on *F*, and *R*- factors based on ALL data will be even larger.

### Fractional atomic coordinates and isotropic or equivalent isotropic displacement parameters (Å^2^)

**Table:**

	*x*	*y*	*z*	*U*_iso_*/*U*_eq_	
P1	0.57919 (3)	−0.04823 (4)	0.124182 (17)	0.02333 (10)	
Na1	0.66932 (5)	0.48279 (7)	0.07453 (3)	0.03971 (17)	
Na2	0.74134 (4)	0.17009 (6)	0.04642 (3)	0.03330 (15)	
O1	0.40206 (9)	0.09598 (13)	0.21979 (6)	0.0394 (3)	
H1	0.4489 (12)	0.098 (2)	0.1958 (9)	0.047*	
O2	0.61673 (8)	−0.09564 (10)	0.20212 (5)	0.0282 (2)	
O3	0.50549 (8)	−0.16097 (12)	0.09628 (5)	0.0350 (2)	
O4	0.52559 (9)	0.08848 (11)	0.12794 (5)	0.0338 (2)	
O5	0.67384 (8)	−0.03767 (11)	0.09123 (5)	0.0318 (2)	
O6	0.89693 (9)	0.06153 (12)	0.03525 (6)	0.0361 (3)	
H2	0.9272 (14)	0.0031 (16)	0.0631 (8)	0.043*	
H3	0.9421 (12)	0.1229 (15)	0.0342 (10)	0.043*	
O7	0.82327 (8)	0.39178 (13)	0.03833 (6)	0.0352 (2)	
H4	0.8329 (15)	0.410 (2)	−0.0014 (4)	0.042*	
H5	0.8815 (8)	0.408 (2)	0.0629 (9)	0.042*	
O8	0.72026 (10)	0.30040 (13)	0.14616 (6)	0.0392 (3)	
H6	0.7723 (11)	0.322 (2)	0.1753 (8)	0.047*	
H7	0.6828 (13)	0.2551 (19)	0.1684 (9)	0.047*	
O9	0.56093 (8)	0.26022 (11)	0.02832 (6)	0.0326 (2)	
H8	0.5482 (14)	0.2058 (16)	0.0587 (7)	0.039*	
H9	0.5390 (14)	0.2237 (19)	−0.0097 (5)	0.039*	
O10	0.77621 (10)	0.69039 (13)	0.08084 (7)	0.0434 (3)	
H10	0.7540 (16)	0.7654 (13)	0.0938 (11)	0.052*	
H11	0.8397 (6)	0.685 (2)	0.0986 (11)	0.052*	
O11	0.53790 (10)	0.58034 (12)	0.13535 (7)	0.0420 (3)	
H12	0.5281 (17)	0.6642 (8)	0.1257 (11)	0.050*	
C1	0.40429 (11)	−0.01837 (17)	0.25947 (8)	0.0318 (3)	
C2	0.30914 (12)	−0.0869 (2)	0.25809 (9)	0.0424 (4)	
H13	0.2496	−0.0541	0.2310	0.051*	
C3	0.30408 (13)	−0.2003 (2)	0.29597 (9)	0.0437 (4)	
H14	0.2409	−0.2441	0.2946	0.052*	
C4	0.39363 (13)	−0.25268 (17)	0.33747 (8)	0.0350 (3)	
C5	0.39111 (16)	−0.37427 (19)	0.37466 (9)	0.0443 (4)	
H15	0.3285	−0.4192	0.3738	0.053*	
C6	0.47905 (18)	−0.4266 (2)	0.41165 (10)	0.0522 (5)	
H16	0.4769	−0.5089	0.4342	0.063*	
C7	0.57301 (17)	−0.3559 (2)	0.41573 (10)	0.0518 (5)	
H17	0.6325	−0.3908	0.4421	0.062*	
C8	0.57852 (13)	−0.23620 (18)	0.38149 (8)	0.0387 (4)	
H18	0.6412	−0.1901	0.3855	0.046*	
C9	0.48927 (11)	−0.18220 (16)	0.34002 (7)	0.0296 (3)	
C10	0.49298 (11)	−0.06224 (15)	0.30036 (7)	0.0273 (3)	
C11	0.59188 (10)	0.01444 (15)	0.30256 (7)	0.0266 (3)	
C12	0.62733 (11)	0.10678 (16)	0.35622 (7)	0.0314 (3)	
C13	0.57337 (15)	0.1248 (2)	0.41085 (9)	0.0479 (4)	
H19	0.5130	0.0760	0.4119	0.057*	
C14	0.60945 (19)	0.2133 (3)	0.46178 (11)	0.0629 (6)	
H20	0.5742	0.2225	0.4977	0.076*	
C15	0.6995 (2)	0.2907 (3)	0.46050 (11)	0.0657 (6)	
H21	0.7224	0.3520	0.4950	0.079*	
C16	0.75278 (17)	0.2760 (2)	0.40923 (11)	0.0552 (5)	
H22	0.8122	0.3276	0.4090	0.066*	
C17	0.71954 (13)	0.18328 (17)	0.35570 (8)	0.0366 (3)	
C18	0.77548 (12)	0.16267 (18)	0.30267 (9)	0.0378 (4)	
H23	0.8356	0.2124	0.3018	0.045*	
C19	0.74264 (11)	0.07110 (16)	0.25277 (8)	0.0324 (3)	
H24	0.7815	0.0563	0.2191	0.039*	
C20	0.64935 (10)	−0.00130 (14)	0.25234 (7)	0.0254 (3)	
C21	0.50869 (18)	0.5732 (2)	0.20062 (11)	0.0533 (5)	
H25A	0.4374	0.6027	0.1974	0.064*	
H25B	0.5520	0.6343	0.2311	0.064*	
C22	0.5196 (2)	0.4332 (3)	0.22760 (13)	0.0656 (6)	
H26A	0.4797	0.3719	0.1963	0.098*	
H26B	0.4952	0.4298	0.2697	0.098*	
H26C	0.5912	0.4069	0.2344	0.098*	

### Atomic displacement parameters (Å^2^)

**Table:**

	*U*^11^	*U*^22^	*U*^33^	*U*^12^	*U*^13^	*U*^23^
P1	0.02712 (18)	0.02280 (18)	0.02071 (17)	0.00115 (12)	0.00595 (13)	0.00013 (12)
Na1	0.0399 (3)	0.0389 (4)	0.0434 (4)	0.0078 (3)	0.0157 (3)	0.0124 (3)
Na2	0.0303 (3)	0.0315 (3)	0.0398 (3)	0.0013 (2)	0.0108 (2)	−0.0015 (2)
O1	0.0335 (6)	0.0470 (7)	0.0395 (6)	0.0102 (5)	0.0115 (5)	0.0121 (5)
O2	0.0382 (5)	0.0252 (5)	0.0213 (4)	0.0023 (4)	0.0055 (4)	0.0011 (4)
O3	0.0374 (6)	0.0348 (6)	0.0310 (5)	−0.0085 (5)	0.0014 (4)	−0.0008 (4)
O4	0.0436 (6)	0.0302 (5)	0.0298 (5)	0.0115 (4)	0.0129 (4)	0.0058 (4)
O5	0.0348 (5)	0.0337 (6)	0.0298 (5)	0.0000 (4)	0.0140 (4)	−0.0013 (4)
O6	0.0301 (5)	0.0311 (6)	0.0471 (7)	0.0012 (4)	0.0069 (5)	0.0030 (5)
O7	0.0312 (5)	0.0442 (7)	0.0317 (5)	−0.0035 (5)	0.0094 (4)	0.0010 (5)
O8	0.0485 (7)	0.0384 (6)	0.0303 (6)	−0.0044 (5)	0.0061 (5)	0.0050 (5)
O9	0.0353 (5)	0.0310 (6)	0.0316 (5)	−0.0027 (4)	0.0060 (4)	0.0041 (4)
O10	0.0425 (6)	0.0415 (7)	0.0460 (7)	0.0006 (5)	0.0072 (5)	−0.0023 (6)
O11	0.0536 (7)	0.0299 (6)	0.0456 (7)	−0.0019 (5)	0.0174 (6)	−0.0030 (5)
C1	0.0301 (7)	0.0380 (8)	0.0288 (7)	0.0010 (6)	0.0090 (6)	0.0006 (6)
C2	0.0279 (7)	0.0583 (11)	0.0403 (9)	−0.0027 (7)	0.0040 (6)	0.0043 (8)
C3	0.0341 (8)	0.0551 (11)	0.0430 (9)	−0.0138 (7)	0.0097 (7)	−0.0037 (8)
C4	0.0441 (8)	0.0363 (8)	0.0269 (7)	−0.0080 (6)	0.0128 (6)	−0.0057 (6)
C5	0.0632 (11)	0.0386 (9)	0.0338 (8)	−0.0153 (8)	0.0160 (8)	−0.0045 (7)
C6	0.0842 (14)	0.0346 (9)	0.0402 (9)	−0.0060 (9)	0.0172 (10)	0.0040 (7)
C7	0.0646 (12)	0.0474 (11)	0.0419 (10)	0.0110 (9)	0.0050 (9)	0.0099 (8)
C8	0.0402 (8)	0.0425 (9)	0.0335 (8)	0.0031 (7)	0.0067 (6)	0.0035 (7)
C9	0.0350 (7)	0.0322 (7)	0.0233 (6)	−0.0012 (6)	0.0100 (5)	−0.0029 (5)
C10	0.0280 (6)	0.0321 (7)	0.0233 (6)	−0.0007 (5)	0.0082 (5)	−0.0026 (5)
C11	0.0265 (6)	0.0290 (7)	0.0240 (6)	0.0011 (5)	0.0038 (5)	0.0011 (5)
C12	0.0334 (7)	0.0329 (8)	0.0274 (7)	0.0030 (6)	0.0036 (6)	−0.0035 (6)
C13	0.0491 (10)	0.0577 (11)	0.0386 (9)	0.0016 (8)	0.0127 (8)	−0.0150 (8)
C14	0.0748 (14)	0.0704 (15)	0.0446 (11)	0.0068 (12)	0.0132 (10)	−0.0261 (10)
C15	0.0786 (15)	0.0637 (14)	0.0491 (12)	0.0000 (12)	−0.0043 (11)	−0.0303 (10)
C16	0.0561 (11)	0.0489 (11)	0.0544 (11)	−0.0094 (9)	−0.0077 (9)	−0.0139 (9)
C17	0.0384 (8)	0.0325 (8)	0.0359 (8)	−0.0015 (6)	−0.0022 (6)	−0.0021 (6)
C18	0.0322 (7)	0.0380 (9)	0.0413 (9)	−0.0078 (6)	0.0014 (6)	0.0058 (7)
C19	0.0301 (7)	0.0378 (8)	0.0305 (7)	−0.0010 (6)	0.0087 (6)	0.0065 (6)
C20	0.0278 (6)	0.0251 (6)	0.0226 (6)	0.0022 (5)	0.0027 (5)	0.0022 (5)
C21	0.0634 (12)	0.0490 (11)	0.0548 (11)	0.0026 (9)	0.0304 (10)	−0.0034 (9)
C22	0.0689 (14)	0.0647 (14)	0.0716 (15)	0.0061 (11)	0.0359 (12)	0.0165 (12)

### Geometric parameters (Å, °)

**Table:**

P1—O5	1.5112 (11)	C3—H14	0.9300
P1—O3	1.5124 (11)	C4—C5	1.414 (2)
P1—O4	1.5231 (11)	C4—C9	1.426 (2)
P1—O2	1.6330 (11)	C5—C6	1.361 (3)
Na1—O8	2.3256 (14)	C5—H15	0.9300
Na1—O6^i^	2.3660 (16)	C6—C7	1.405 (3)
Na1—O7	2.4347 (14)	C6—H16	0.9300
Na1—O10	2.4645 (16)	C7—C8	1.371 (3)
Na1—O11	2.4773 (15)	C7—H17	0.9300
Na1—O9	2.6842 (15)	C8—C9	1.418 (2)
Na2—O6	2.3484 (14)	C8—H18	0.9300
Na2—O8	2.4423 (15)	C9—C10	1.430 (2)
Na2—O7	2.4453 (15)	C10—C11	1.494 (2)
Na2—O5	2.4581 (14)	C11—C20	1.374 (2)
Na2—O9	2.4937 (14)	C11—C12	1.427 (2)
Na2—O10^ii^	2.5483 (17)	C12—C17	1.426 (2)
O1—C1	1.377 (2)	C12—C13	1.422 (2)
O1—H1	0.847 (17)	C13—C14	1.366 (3)
O2—C20	1.3850 (17)	C13—H19	0.9300
O6—H2	0.852 (16)	C14—C15	1.408 (4)
O6—H3	0.848 (16)	C14—H20	0.9300
O7—H4	0.853 (11)	C15—C16	1.354 (3)
O7—H5	0.851 (14)	C15—H21	0.9300
O8—H6	0.847 (16)	C16—C17	1.423 (2)
O8—H7	0.847 (18)	C16—H22	0.9300
O9—H8	0.852 (15)	C17—C18	1.415 (3)
O9—H9	0.851 (12)	C18—C19	1.363 (2)
O10—H10	0.850 (15)	C18—H23	0.9300
O10—H11	0.849 (12)	C19—C20	1.414 (2)
O11—C21	1.437 (2)	C19—H24	0.9300
O11—H12	0.851 (9)	C21—C22	1.476 (3)
C1—C10	1.374 (2)	C21—H25A	0.9700
C1—C2	1.414 (2)	C21—H25B	0.9700
C2—C3	1.359 (3)	C22—H26A	0.9600
C2—H13	0.9300	C22—H26B	0.9600
C3—C4	1.416 (3)	C22—H26C	0.9600
			
Na1···Na2	3.2900 (13)	Na2···H3	2.728 (17)
Na1···Na2^i^	3.4236 (12)	Na2···H8	2.612 (18)
			
O5—P1—O3	114.03 (7)	C10—C1—O1	122.37 (14)
O5—P1—O4	112.55 (6)	C10—C1—C2	121.02 (15)
O3—P1—O4	113.11 (7)	O1—C1—C2	116.57 (14)
O5—P1—O2	108.12 (6)	C3—C2—C1	120.54 (16)
O3—P1—O2	102.51 (6)	C3—C2—H13	119.7
O4—P1—O2	105.51 (6)	C1—C2—H13	119.7
O8—Na1—O6^i^	148.58 (5)	C2—C3—C4	120.93 (15)
O8—Na1—O7	74.98 (5)	C2—C3—H14	119.5
O6^i^—Na1—O7	91.55 (5)	C4—C3—H14	119.5
O8—Na1—O10	120.34 (5)	C3—C4—C5	121.83 (16)
O6^i^—Na1—O10	83.76 (5)	C3—C4—C9	118.65 (15)
O7—Na1—O10	79.89 (5)	C5—C4—C9	119.49 (16)
O8—Na1—O11	97.96 (5)	C6—C5—C4	120.94 (18)
O6^i^—Na1—O11	99.48 (5)	C6—C5—H15	119.5
O7—Na1—O11	167.51 (5)	C4—C5—H15	119.5
O10—Na1—O11	95.38 (5)	C5—C6—C7	119.82 (18)
O8—Na1—O9	70.56 (5)	C5—C6—H16	120.1
O6^i^—Na1—O9	81.61 (4)	C7—C6—H16	120.1
O7—Na1—O9	90.80 (5)	C8—C7—C6	121.10 (18)
O10—Na1—O9	162.42 (5)	C8—C7—H17	119.5
O11—Na1—O9	96.58 (5)	C6—C7—H17	119.5
O6—Na2—O8	122.83 (5)	C7—C8—C9	120.48 (17)
O6—Na2—O7	89.87 (5)	C7—C8—H18	119.8
O8—Na2—O7	72.74 (5)	C9—C8—H18	119.8
O6—Na2—O5	91.94 (5)	C8—C9—C4	118.09 (15)
O8—Na2—O5	91.78 (5)	C8—C9—C10	122.24 (14)
O7—Na2—O5	162.52 (5)	C4—C9—C10	119.66 (14)
O6—Na2—O9	164.98 (5)	C1—C10—C9	119.15 (14)
O8—Na2—O9	72.19 (4)	C1—C10—C11	120.03 (14)
O7—Na2—O9	95.26 (5)	C9—C10—C11	120.82 (13)
O5—Na2—O9	87.40 (4)	C20—C11—C12	118.94 (13)
O6—Na2—O10^ii^	82.29 (5)	C20—C11—C10	120.45 (13)
O8—Na2—O10^ii^	141.97 (5)	C12—C11—C10	120.60 (13)
O7—Na2—O10^ii^	80.01 (4)	C17—C12—C13	118.56 (15)
O5—Na2—O10^ii^	117.47 (5)	C17—C12—C11	119.58 (14)
O9—Na2—O10^ii^	84.71 (5)	C13—C12—C11	121.87 (15)
C1—O1—H1	113.9 (15)	C14—C13—C12	120.6 (2)
C20—O2—P1	121.22 (9)	C14—C13—H19	119.7
P1—O5—Na2	126.55 (6)	C12—C13—H19	119.7
Na2—O6—Na1^ii^	93.14 (5)	C13—C14—C15	120.7 (2)
Na2—O6—H2	123.8 (14)	C13—C14—H20	119.6
Na1^ii^—O6—H2	116.8 (15)	C15—C14—H20	119.6
Na2—O6—H3	107.7 (14)	C16—C15—C14	120.21 (18)
Na1^ii^—O6—H3	110.7 (14)	C16—C15—H21	119.9
H2—O6—H3	104.2 (19)	C14—C15—H21	119.9
Na1—O7—Na2	84.78 (4)	C15—C16—C17	121.2 (2)
Na1—O7—H4	118.2 (14)	C15—C16—H22	119.4
Na2—O7—H4	112.7 (14)	C17—C16—H22	119.4
Na1—O7—H5	117.6 (14)	C18—C17—C12	118.90 (14)
Na2—O7—H5	119.4 (14)	C18—C17—C16	122.43 (17)
H4—O7—H5	104.1 (19)	C12—C17—C16	118.66 (17)
Na1—O8—Na2	87.23 (5)	C19—C18—C17	121.02 (14)
Na1—O8—H6	110.2 (15)	C19—C18—H23	119.5
Na2—O8—H6	120.8 (15)	C17—C18—H23	119.5
Na1—O8—H7	127.1 (14)	C18—C19—C20	119.86 (14)
Na2—O8—H7	108.8 (15)	C18—C19—H24	120.1
H6—O8—H7	104 (2)	C20—C19—H24	120.1
Na2—O9—Na1	78.81 (4)	C11—C20—O2	118.46 (12)
Na2—O9—H8	88.3 (13)	C11—C20—C19	121.64 (13)
Na1—O9—H8	114.7 (13)	O2—C20—C19	119.82 (13)
Na2—O9—H9	98.4 (13)	O11—C21—C22	111.06 (17)
Na1—O9—H9	137.1 (14)	O11—C21—H25A	109.4
H8—O9—H9	107.9 (19)	C22—C21—H25A	109.4
Na1—O10—Na2^i^	86.13 (5)	O11—C21—H25B	109.4
Na1—O10—H10	120.7 (15)	C22—C21—H25B	109.4
Na2^i^—O10—H10	113.8 (16)	H25A—C21—H25B	108.0
Na1—O10—H11	118.5 (15)	C21—C22—H26A	109.5
Na2^i^—O10—H11	109.3 (16)	C21—C22—H26B	109.5
H10—O10—H11	107 (2)	H26A—C22—H26B	109.5
C21—O11—Na1	139.11 (12)	C21—C22—H26C	109.5
C21—O11—H12	101.7 (16)	H26A—C22—H26C	109.5
Na1—O11—H12	110.5 (15)	H26B—C22—H26C	109.5
			
O5—P1—O2—C20	85.95 (11)	O9—Na1—O11—C21	88.50 (18)
O3—P1—O2—C20	−153.29 (10)	C10—C1—C2—C3	−1.8 (3)
O4—P1—O2—C20	−34.69 (12)	O1—C1—C2—C3	−179.66 (16)
O3—P1—O5—Na2	130.38 (8)	C1—C2—C3—C4	−0.2 (3)
O4—P1—O5—Na2	−0.19 (10)	C2—C3—C4—C5	−176.76 (17)
O2—P1—O5—Na2	−116.33 (7)	C2—C3—C4—C9	1.4 (3)
O6—Na2—O5—P1	168.16 (8)	C3—C4—C5—C6	176.72 (17)
O8—Na2—O5—P1	45.22 (8)	C9—C4—C5—C6	−1.5 (3)
O7—Na2—O5—P1	72.42 (18)	C4—C5—C6—C7	3.1 (3)
O9—Na2—O5—P1	−26.85 (8)	C5—C6—C7—C8	−1.8 (3)
O10^ii^—Na2—O5—P1	−109.52 (8)	C6—C7—C8—C9	−1.1 (3)
O8—Na2—O6—Na1^ii^	−178.06 (5)	C7—C8—C9—C4	2.7 (2)
O7—Na2—O6—Na1^ii^	−108.82 (5)	C7—C8—C9—C10	−176.16 (16)
O5—Na2—O6—Na1^ii^	88.57 (5)	C3—C4—C9—C8	−179.63 (15)
O9—Na2—O6—Na1^ii^	1.4 (2)	C5—C4—C9—C8	−1.4 (2)
O10^ii^—Na2—O6—Na1^ii^	−28.89 (5)	C3—C4—C9—C10	−0.8 (2)
O8—Na1—O7—Na2	45.99 (4)	C5—C4—C9—C10	177.46 (14)
O6^i^—Na1—O7—Na2	−105.25 (4)	O1—C1—C10—C9	−179.85 (13)
O10—Na1—O7—Na2	171.38 (5)	C2—C1—C10—C9	2.4 (2)
O11—Na1—O7—Na2	102.7 (2)	O1—C1—C10—C11	−0.4 (2)
O9—Na1—O7—Na2	−23.62 (4)	C2—C1—C10—C11	−178.07 (15)
O6—Na2—O7—Na1	−168.48 (5)	C8—C9—C10—C1	177.67 (15)
O8—Na2—O7—Na1	−43.84 (4)	C4—C9—C10—C1	−1.1 (2)
O5—Na2—O7—Na1	−72.42 (15)	C8—C9—C10—C11	−1.8 (2)
O9—Na2—O7—Na1	25.66 (4)	C4—C9—C10—C11	179.38 (13)
O10^ii^—Na2—O7—Na1	109.33 (5)	C1—C10—C11—C20	−78.74 (19)
O6^i^—Na1—O8—Na2	21.43 (11)	C9—C10—C11—C20	100.74 (17)
O7—Na1—O8—Na2	−45.89 (4)	C1—C10—C11—C12	100.25 (17)
O10—Na1—O8—Na2	−114.32 (6)	C9—C10—C11—C12	−80.28 (18)
O11—Na1—O8—Na2	144.64 (5)	C20—C11—C12—C17	1.8 (2)
O9—Na1—O8—Na2	50.43 (4)	C10—C11—C12—C17	−177.24 (14)
O6—Na2—O8—Na1	124.57 (5)	C20—C11—C12—C13	−178.02 (15)
O7—Na2—O8—Na1	46.30 (4)	C10—C11—C12—C13	3.0 (2)
O5—Na2—O8—Na1	−141.97 (5)	C17—C12—C13—C14	−0.2 (3)
O9—Na2—O8—Na1	−55.27 (4)	C11—C12—C13—C14	179.57 (19)
O10^ii^—Na2—O8—Na1	0.10 (9)	C12—C13—C14—C15	1.4 (4)
O6—Na2—O9—Na1	−133.02 (18)	C13—C14—C15—C16	−1.4 (4)
O8—Na2—O9—Na1	46.47 (4)	C14—C15—C16—C17	0.2 (4)
O7—Na2—O9—Na1	−23.50 (4)	C13—C12—C17—C18	177.98 (16)
O5—Na2—O9—Na1	139.18 (4)	C11—C12—C17—C18	−1.8 (2)
O10^ii^—Na2—O9—Na1	−102.92 (4)	C13—C12—C17—C16	−1.0 (2)
Na1^ii^—Na2—O9—Na1	−131.92 (3)	C11—C12—C17—C16	179.20 (16)
O8—Na1—O9—Na2	−50.24 (4)	C15—C16—C17—C18	−177.9 (2)
O6^i^—Na1—O9—Na2	114.95 (4)	C15—C16—C17—C12	1.0 (3)
O7—Na1—O9—Na2	23.51 (4)	C12—C17—C18—C19	−0.2 (2)
O10—Na1—O9—Na2	81.01 (16)	C16—C17—C18—C19	178.72 (17)
O11—Na1—O9—Na2	−146.40 (5)	C17—C18—C19—C20	2.3 (2)
O8—Na1—O10—Na2^i^	131.19 (5)	C12—C11—C20—O2	177.15 (12)
O6^i^—Na1—O10—Na2^i^	−27.34 (4)	C10—C11—C20—O2	−3.9 (2)
O7—Na1—O10—Na2^i^	65.36 (4)	C12—C11—C20—C19	0.3 (2)
O11—Na1—O10—Na2^i^	−126.32 (5)	C10—C11—C20—C19	179.30 (13)
O9—Na1—O10—Na2^i^	6.41 (17)	P1—O2—C20—C11	115.23 (13)
O8—Na1—O11—C21	17.30 (18)	P1—O2—C20—C19	−67.86 (16)
O6^i^—Na1—O11—C21	171.05 (18)	C18—C19—C20—C11	−2.3 (2)
O7—Na1—O11—C21	−37.4 (3)	C18—C19—C20—O2	−179.16 (13)
O10—Na1—O11—C21	−104.40 (18)	Na1—O11—C21—C22	−38.2 (3)

Symmetry codes: (i) −*x*+3/2, *y*+1/2, −*z*; (ii) −*x*+3/2, *y*−1/2, −*z*.

### Hydrogen-bond geometry (Å, °)

**Table:**

*D*—H···*A*	*D*—H	H···*A*	*D*···*A*	*D*—H···*A*
O1—H1···O4	0.85 (2)	1.84 (2)	2.6687 (19)	166 (2)
O6—H2···O11^iii^	0.85 (2)	2.04 (2)	2.854 (2)	159 (2)
O6—H3···O9^iii^	0.85 (2)	1.96 (2)	2.7958 (19)	171 (2)
O7—H4···O5^i^	0.85 (1)	1.87 (1)	2.7136 (18)	168 (2)
O7—H5···O4^iii^	0.85 (1)	2.11 (1)	2.9473 (19)	170 (2)
O8—H6···O1^iii^	0.85 (2)	1.96 (2)	2.775 (2)	163 (2)
O9—H8···O4	0.85 (2)	1.88 (2)	2.7259 (18)	178 (1)
O9—H9···O3^iv^	0.85 (1)	1.85 (1)	2.6978 (18)	174 (2)
O10—H10···O5^v^	0.85 (2)	2.20 (2)	3.012 (2)	161 (2)
O10—H11···O3^iii^	0.85 (1)	2.20 (1)	2.984 (2)	154 (2)
O11—H12···O3^v^	0.85 (1)	1.82 (1)	2.6711 (19)	174 (2)

Symmetry codes: (iii) *x*+1/2, −*y*+1/2, *z*; (i) −*x*+3/2, *y*+1/2, −*z*; (iv) −*x*+1, −*y*, −*z*; (v) *x*, *y*+1, *z*.
